# Isoform dependent regulation of human HCN channels by cholesterol

**DOI:** 10.1038/srep14270

**Published:** 2015-09-25

**Authors:** Oliver Fürst, Nazzareno D’Avanzo

**Affiliations:** 1 From the Département de physiologie moléculaire et intégrative, Université de Montréal and the Groupe d’Étude des Protéines Membranaires (GÉPROM), 2960 Chemin de la Tour, Montreal, Quebec, H3T 1J4.

## Abstract

Cholesterol has been shown to regulate numerous ion channels. HCN channels represent the molecular correlate of I_f_ or I_h_ in sinoatrial node (SAN) and neuronal cells. Previous studies have implicated a role for cholesterol in the regulation of rabbit HCN4 channels with effects on pacing in the rabbit SAN. Using electrophysiological and biochemical approaches, we examined the effect of cholesterol modulation on human HCN1, HCN2 and HCN4 isoforms. Patch-clamp experiments uncovered isoform specific differences in the effect of cholesterol on gating kinetics upon depletion by MβCD or mevastatin or enrichment using MβCD/cholesterol. Most dramatically cholesterol had isoform specific effects on mode-shifting, which has been suggested to play a key role in stabilizing firing rate and preventing arrhythmic firing in SAN cells and neurons. Mode-shifting in HCN1 channels was insensitive to cholesterol manipulation, while HCN2 and HCN4 were strongly affected. Trafficking of each isoform to the plasma membrane was also affected by cholesterol modulation differentially between isoforms, however, each isoform remained localized in lipid raft domains after cholesterol depletion. These effects may contribute to the side effects of cholesterol reducing therapies including disrupted heart rhythm and neuropathic pain, as well as the susceptibility of sinus dysfunction in patients with elevated cholesterol.

The action potential of a sinoatrial node (SAN) cell is characterized by the presence of a progressive diastolic depolarization between −65 mV and −45 mV. Although the diastolic depolarization results from the concerted action of several currents, I_h_, which was identified in the late 1970s, serves as a primary initiator. Hyperpolarization activated cyclic-nucleotide gated (HCN) channels represent the molecular correlate of the currents I_h_ or I_f_ in SAN and neuronal cells. The sensitivity of these channels to cyclic-nucleotides enables I_h_ to adjust to stimulation of the autonomic nervous system.

Four mammalian isoforms (HCN1-HCN4) exist, sharing approximately 60% sequence identity. In all mammals examined to date, HCN4 is the principle component of I_h_ in the SAN^1^,^2^,^3^,^4^,^5^. The expression of other isoforms is significantly weaker, and species dependent^3^,^4^. SAN cells of HCN4 deficient mice have a 70–80% reduction in I_h_^6^, while HCN2 channels contribute the remaining 20–30%^7^. Moreover, HCN4^*–/–*^ deletion resulted in embryonic death in mice due to a failure to generate mature pacemaking SAN cells^7^,^8^, while HCN2 deficient mice display only mild sinus dysrhythmia at rest^7^. Non-pacemaking cardiomyocytes of the atria and ventricles also express HCN channels, with their function in these cells yet to be conclusively determined. However, increased I_h_ in ventricular myocytes has been reported in cardiac diseases such as hypertrophy, ischemic cardiomyopathy, and heart failure^9^,^10^,^11^,^12^,^13^. Also, the addition of the HCN channel specific inhibitor ivabradine to standard therapy reduced the rates of hospital admissions and cardiovascular death in heart failure patients examined during a large clinical trial (Systolic Heart Failure Treatment with the I_f_ Inhibitor Ivabradine Trial, SHIFT)^14^,^15^. Thus, understanding the regulation of HCN channels is an important factor for understanding cardiac and neuronal function and the consequences of various therapeutic approaches.

Topologically, HCN channels are members of the pore-loop cation channel superfamily, with each subunit containing 6 transmembrane α-helices (S1–S6), a re-entrant loop between the S5 and S6 helices that forms the selectivity filter, and a C-terminal cyclic-nucleotide binding domain (CNBD) attached to the S6 via an 80 amino acid C-linker. Channels are formed by homo- or hetero-tetrameric assembly of the subunits^16^. Electrophysiological recordings of HCN channels have characteristic properties, including activation with sigmoidal kinetics upon membrane hyperpolarization, a lack of voltage-dependent inactivation, conduction of Na^+^ and K^+^, a shift in the activation curve as a result of direct interaction with cAMP and cGMP, and inhibition by millimolar concentrations of external Cs^+^^17^. The activation kinetics of the four mammalian isoforms vary by several fold, and differ from one another in their response to cyclic nucleotides. cAMP shifts the voltage-dependence of activation in HCN2 and HCN4 by +15 mV, while HCN1 and HCN3 are only weakly modulated by cAMP^2^,^18^,^19^,^20^.

The activity of HCN channels have been recently shown to be regulated by membrane lipids. Voltage-dependent gating of HCN channels is allosterically regulated by phosphoinositides (particularly PIP_2_ but not PI), phosphatidic acid (PA), and the fatty acid arachidonic acid (AA)^21^,^22^,^23^,^24^. This regulation appears independent of the action of cAMP, since their effects are still observed in channels lacking the CNBD^23^,^24^. Cholesterol, the major sterol in all mammalian plasma membranes, has been implicated in the modulation of the function of various ion channels^25^. Cholesterol content in the sarcolemma of cardiac myocytes has been shown to increase when serum cholesterol levels are elevated^26^, increasing nearly 20% in diabetes^27^. A recent study has indicated that cholesterol depletion by MβCD in HEK cells and ventricular myocytes impaired rabbit HCN4 channel localization into lipid rafts and shifted V_1/2_ of activation to more positive potentials and increased diastolic depolarization in rabbit SAN cells^28^. In this study, we systematically explore the regulation of the three human cardiac HCN isoforms (HCN1, HCN2, and HCN4) by membrane cholesterol.

## Materials and Methods

### Cell culture

CHO-K1 cells (ATCC, Manassas, VA) were cultured at 37°C, 5% CO_2_ in F12K Eagle’s medium supplemented with 10% (v/v) fetal bovine serum (FBS) and 1% Penicillin/Streptomycin. Cells were transfected with 4 μg of human HCN1, HCN2 or HCN4 as well as 750 ng of eGFP using Lipofectamine 2000 (Life Technologies, Carlsbad, CA) in serum-free OptiMEM (Sigma-Aldrich, St. Louis, MO), and returned to supplemented F12K after 3-4 hrs. Transfected cells were incubated in supplemented culture media for 24–48 hours prior to the electrophysiological recordings or biochemical experiments. Cholesterol depletion was accomplished by exposing cells to 5 mM Methyl-*β*-cyclodextrin (M*β*CD) for 60 min as previously performed^29^,^30^ or by inhibition of the cholesterol synthesis pathway by culturing cells in F12K media supplemented with 10% LPDS and 30 μM mevastatin. Cholesterol enrichment was achieved by incubating cells for 30 min with 5 mM M*β*CD pre-saturated with cholesterol (Sigma-Aldrich). Efficacy of the treatments were quantified, using the Amplex Red cholesterol assay kit (Life Technologies) to quantify the cholesterol content in the cell membrane. After 30–60 min fluorescence from the reaction was read using an Infinite® 200 Pro plate reader (Tecan Group Ltd., Männedorf, Switzerland).

### Preparation of Lipoprotein-deficient serum (LPDS)

Lipoprotein-deficient serum (LPDS) was prepared following the protocol of Renaud *et al.*^31^ with slight variation. Briefly, FBS was adjusted to a density of 1.215 g/mL by adding KBr. After overlaying the FBS with a KBr solution at the same density, the mixture was centrifuged for 65 h at 235,500 g at 4 °C. The floating lipoproteins were removed and the remaining serum was dialysed against 6 changes of 4L Phosphate-buffer saline (PBS) pH 7.4 at 4 °C.

### Membrane fractioning by discontinuous sucrose gradient

24–30 h post-transfection, cells were washed thrice with PBS and scraped into Na_2_CO_3_ pH 11 and left on ice for 20 min. The solution was sonicated thrice for 20 sec bursts. By adding an equal volume of 90% sucrose/MES/NaCl-Buffer, the solution was adjusted to 45% sucrose density. Layers of 35% and 5% sucrose were then cautiously added on top of the lysate. All samples were centrifuged at 273,000 g for 16 hours at 4 °C (SW60 rotor, Beckman Instruments, Palo Alto, CA). 12 fractions of equal volumes (1 mL) were collected and their protein content quantified by Nanodrop. The content of cholesterol for each fraction was assayed by the Amplex Red Assay (Life Technologies, Carlsbad, CA) according to the manufacturer’s instructions. Notably, low-density fractions 1–3 usually contain little to no protein, and therefore these samples were excluded from use during further experiments.

### Cell surface biotinylation

Cells were washed thrice with ice-cold PBS and then incubated for 30 min at 4 °C with 1 mM EZ-link sulfo-N-hydroxysuccinimide (sulfo-NHS)-SS-biotin (Pierce, Rockford, IL). After rinsing the cells twice with PBS-glycine, the cells were scraped into a lysis buffer (0.1% IGEPAL, 1% SDS, 250 mM NaCl, 50 mM Tris-HCl pH 7.5 and protease inhibitor) and incubated for 30 min at 4 °C. Three 20 secs bursts of sonication ensured complete cell rupturing. After a 30 min centrifugation at 20,800 g the protein in the supernatant was determined and 800 mg of protein was incubated overnight at 4 °C with immobilized Streptavidin (Pierce). After washing the resin at least seven times with binding buffer (PBS, 0.1% IGEPAL, 0.1% SDS), the biotinylated proteins were eluted by Laemmli buffer containing 0.5 M DTT. Samples were then probed by western blot using isoform specific anti-HCN antibodies.

### Western blotting and densitometric analysis

Since the expression several of candidates for internal controls, such as Na/K ATPase, have also been shown to be modulated by MβCD^32^, protein content in each faction was quantified in each fraction by assessing absorption at λ = 280 nm, and normalizing before loading on an SDS/PAGE. Protein samples were separated by SDS-PAGE on 8% polyacrylamide gels and transferred onto a PVDF membrane (BioRad). The blots were blocked with 5% milk and probed with rabbit anti-HCN antibody (1:500, Alomone labs, Jerusalem, Isreal) followed by a horseradish-peroxidase-conjugated secondary antibody (1:10,000, Santa Cruz Biotechnology, Santa Cruz, CA). HCN and Caveolin-1 bands were visualized using a peroxidase-based chemiluminescent detection kit (Pierce, Rockford, IL) and quantitated using ImageJ software (NIH).

### Electrophysiology

Whole-cell currents were recorded from CHO-K1 cells transfected with HCN1, HCN2, or HCN4 channels 24–48 hours post-transfection. Glass pipettes were pulled to a final resistance of 2–4 MΩ. The external and internal solution were symmetrical and contained (in mM): 150 KCl, 10 HEPES pH 7.3, 2 MgCl_2_ and 1 EGTA. All recordings were performed after 2 mins of dialyzing the internal solution following membrane rupture in order to avoid issues of current rundown. Data were collected at 22–25 °C at 10 kHz with a 1 kHz low-pass Bessel filter using a conventional Axopatch 200B Amplifier and Digidata 1440A digitizer. Capacitance and series resistance were electronically compensated. Activation was assessed by stepping to voltages between −160 mV and −40 mV (Δ + 10 mV) from a holding potential (V_H_) of 0 mV, followed by a step to +30 mV. Steady-state activation curves were assessed from the peak of the tail currents. Non-equilibrium experiments involved a pre-pulse to −70 mV prior to the activation steps for a duration of 100 ms, 500 ms, or 1000 ms for HCN1, HCN2, and HCN4 respectively. Deactivation was assessed by a pre-pulse to −130 mV followed by test pulses from +50 mV to −60 mV (Δ-10 mV). Hysteresis was also assessed by ramps from 0 mV to −150 mV and back at varying speeds. For every protocol, each test pulse was followed by a 17–24 s interpulse interval at V_H_ to ensure complete channel deactivation. Data were analyzed using pClamp 10 and Origin8.0 software packages.

## Results

### Biophysical properties of HCN1, 2 and 4 after manipulating cellular cholesterol content

To study the effect of cholesterol content on human HCN channel activity, we first verified the effect of treatment on cholesterol content in CHO-K1 cells. Treatment with either 5 mM methyl-beta-cyclodextrin (MβCD) or 30 μM mevastatin (an HMG-CoA reductase inhibitor) had nearly equivalent effects on decreasing cholesterol content by 40–60% compared to untreated (control) cells, while treatment with MβCD pre-complexed with cholesterol (MβCD/cholesterol) increased membrane cholesterol by nearly 50% (Supp. Fig. 1).

**Figure 1:**
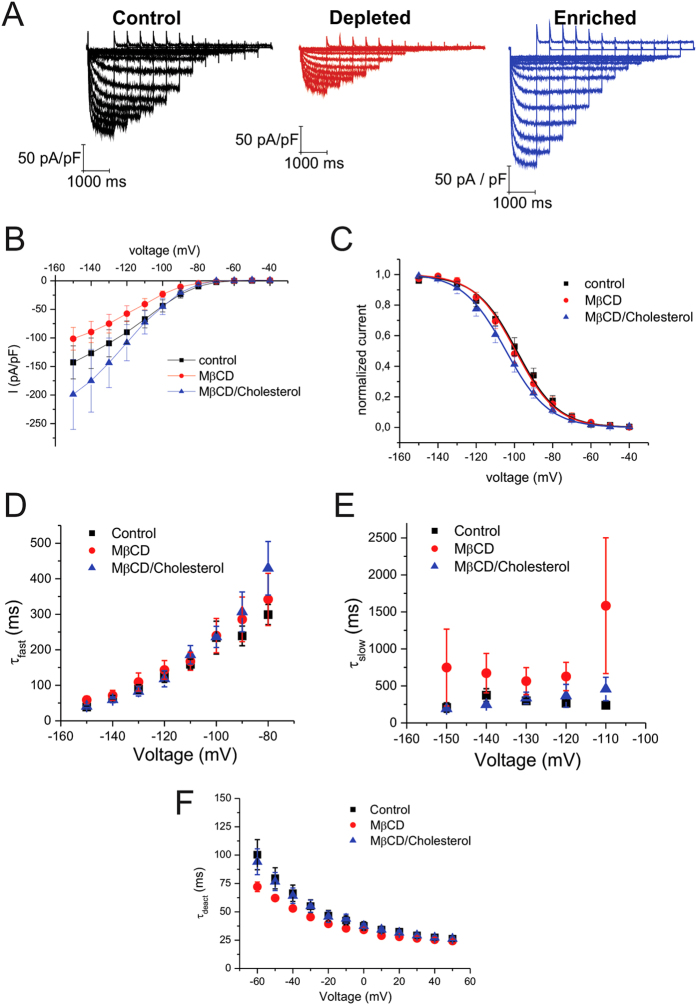
Regulation of HCN1 by cholesterol. (**A**) Representative HCN1 current traces from cells that underwent cholesterol depletion by MβCD (red) or enrichment by MβCD**/**cholesterol (blue) were compared to control (black). (**B**) Current densities of HCN1 are reduced upon cholesterol depletion and unchanged with enrichment. (**C**) Steady-state activation was not affected by modification of membrane cholesterol content. (**D**) HCN1 channel activation can be fit by a dual-exponential function. The fast component of HCN1 channel activation was unaffected by cholesterol modification, however, **(E)** the slow component was slower by nearly 2-fold upon cholesterol depletion. (**F**) The kinetics of HCN1 deactivation were unaffected by cholesterol manipulation. (n > 6; P < 0.05).

Treatment of CHO-K1 cells expressing human HCN1 channels by MβCD resulted in reduced current density compared to untreated control cells (Fig. 1A,B). To verify that this effect was specific to the effects of membrane cholesterol, and not due to unspecific effects of MβCD, we also examined the effect of 30 μM mevastatin, which blocks cholesterol synthesis. Similar to the effect of MβCD, current densities were also reduced in cells treated with mevastatin. While there was a trend towards an increase in the current density with the enrichment of cellular cholesterol by MβCD/cholesterol (P = 0.12), statistical significance could not be resolved. Both depletion and enrichment had no effect on the steady-state activation properties of HCN1 channels (Fig. 1C; Table 1). HCN1 activation currents can be described by a dual-exponential function, whose fast time component (τ_fast_) was unchanged by modulation of membrane cholesterol (Fig. 1D), however, cholesterol depletion reduced the slow component of activation (τ_slow_) by 2-fold (Fig. 1E). No observable effect on HCN1 deactivation kinetics could be discerned.

**Figure 2:**
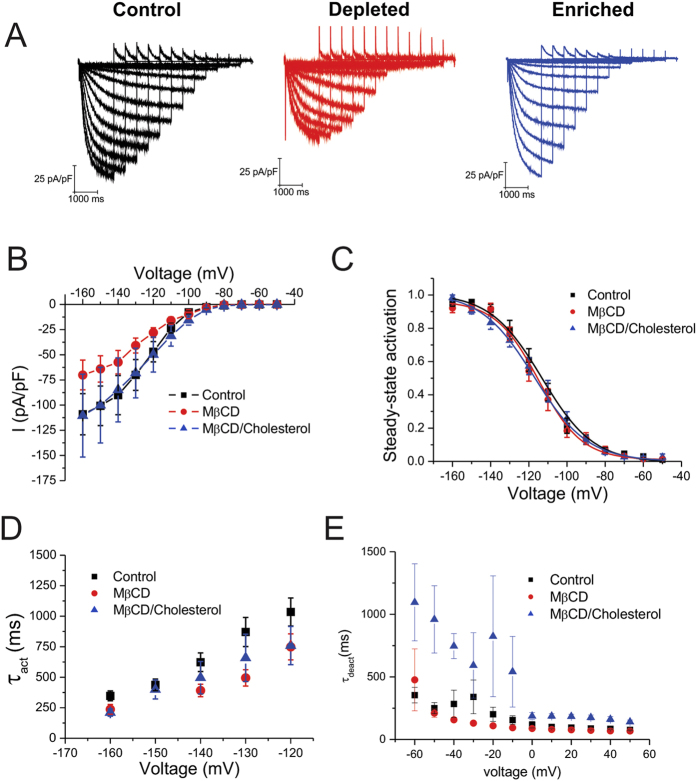
Regulation of HCN2 by cholesterol. (**A**) Representative HCN2 current traces from cells that underwent cholesterol depletion by MβCD (red) or enrichment by MβCD**/**cholesterol (blue) were compared to control (black). (**B**) Current densities of HCN2 are reduced upon cholesterol depletion and unchanged with enrichment. (**C**) Steady-state activation was not affected by modification of membrane cholesterol content. (**D**) HCN2 channel activation can be fit by a single-exponential function which was unaffected by cholesterol modification. (**E**) HCN2 deactivation can also be described by a single-exponential function whose rate decreased upon cholesterol enrichment and was unchanged upon depletion. (n > 10; P < 0.05).

**Figure 3:**
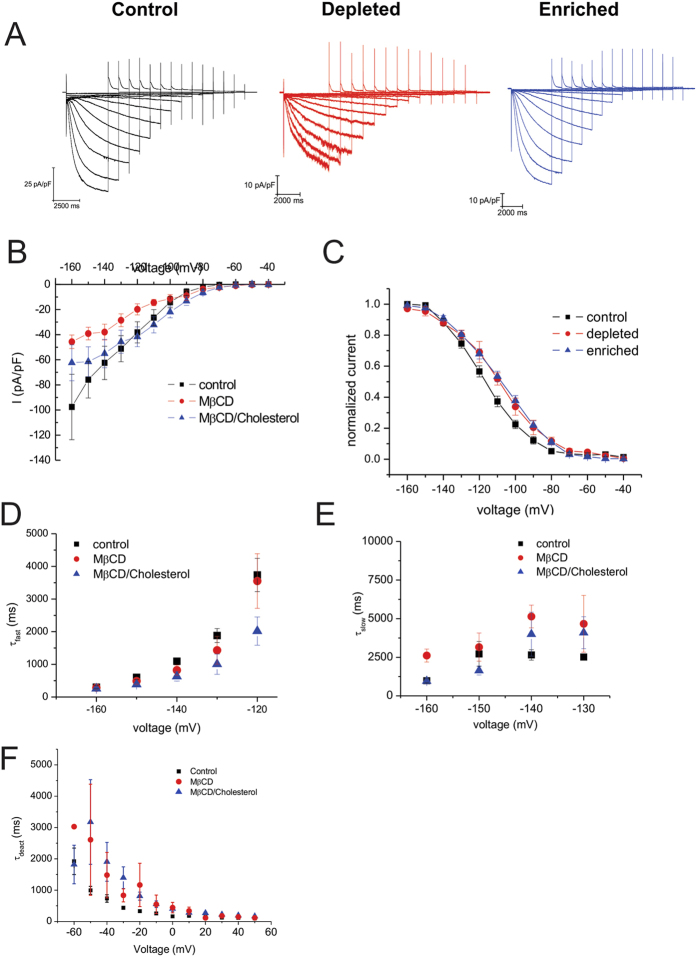
Regulation of HCN4 by cholesterol. (**A**) Representative HCN4 current traces from cells that underwent cholesterol depletion by MβCD (red) or enrichment by MβCD**/**cholesterol (blue) were compared to control (black). (**B**) Current densities of HCN4 are reduced upon cholesterol depletion and unchanged with enrichment. (**C**) Steady-state activation was not affected by modification of membrane cholesterol content. (**D,E**) HCN4 channel activation can be fit by a dual-exponential function whose 2 components were unchanged compared to control. (**F**) The kinetics of HCN4 deactivation can be described by mono-exponential functions which were slowed with cholesterol depletion and enrichment. (n > 8; P < 0.05).

To determine if the effect of cholesterol modulation on HCN1 channels could be generalized to other human HCN channel isoforms, we further examined the effects on the other cardiac isoforms, HCN2 and HCN4 (Figs 2 and 3). Intriguingly, we observed differential effects of cholesterol modulation on these isoforms. Similar to HCN1, both HCN2 and HCN4 channels showed a decrease in current density upon cholesterol depletion by either MβCD (Fig. 2A,B; Fig. 3A,B) or mevastatin (Supp. Fig. 1B,C). Moreover, current densities in cells expressing these isoforms enriched with cholesterol remained similar to control (Fig. 2A,B; Fig. 3A,B). HCN2 channels showed no differences in steady-state activation properties with changes in cholesterol content, however, steady-state properties of HCN4 channels were shifted approximately +10 mV by either cholesterol depletion or enrichment (Fig. 3C; Table 1). Tail currents were too small in HCN4 expressing cells treated with mevastatin to reliably enable us to determine steady-state activation properties. Intriguingly, the effects of cholesterol modulation on human HCN2 and HCN4 kinetics differed from our observations in human HCN1 channels. The activation kinetics of HCN2 and HCN4 channels were unaffected by cholesterol modulation (Figs 2D and 3D,E). However, unlike HCN1 channels, the deactivation kinetics of HCN2 and HCN4 channels were slowed by cholesterol enrichment (Figs 2E and 3F). These data suggest the effect of cholesterol on HCN channels is isoform specific.

### Effect of cholesterol modulation on HCN channel distribution and trafficking.

**Figure 4:**
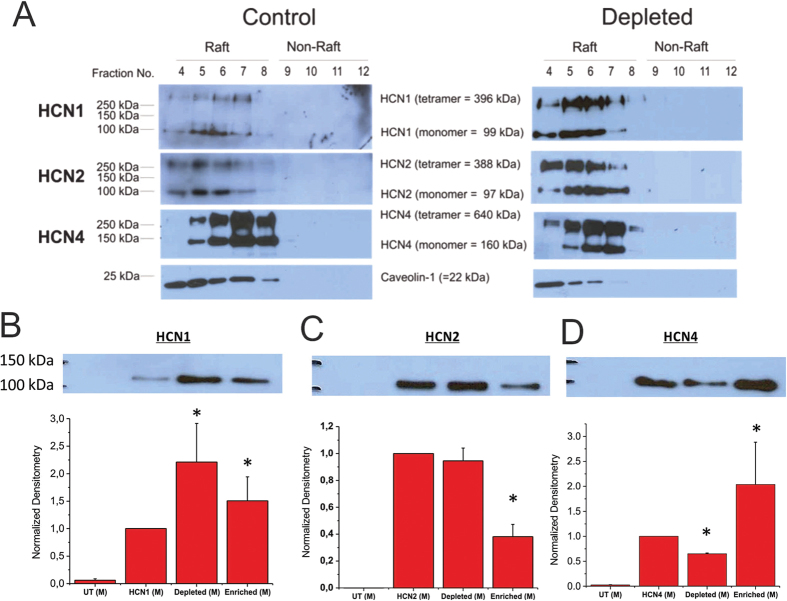
Distribution of HCN channels in CHO-K1 cell membranes. (**A**) HCN1, HCN2 and HCN4 channels were all found to be distributed into low-density fractions typically associated with lipid-raft domains isolated by discontinuous sucrose gradient methods. The distribution of these channels was unchanged upon treatment with MβCD to deplete membranes of cholesterol. (n = 3). Biotinylation assays were used to assess the effect of cholesterol modulation HCN channel trafficking to the membrane **(B–D)**. We observed isoform dependent effects of on trafficking upon cholesterol depletion by MβCD or enrichment by MβCD**/**cholesterol. (n = 3; P < 0.05).

Cholesterol is capable of self-aggregating into low density domains in which numerous channels have been shown to associate^33^. In some cases, disruption of these raft-like domains leads to a redistribution of channels into higher density lipid fractions and altered protein function^29^. Rabbit HCN4 channels have been suggested to reside in cholesterol rich domains^28^, while this has not been examined in human HCN channels. We determined that human HCN1, HCN2, and HCN4 channels are all expressed in low-density cholesterol rich membrane fractions, isolated by discontinuous sucrose gradient (Fig. 4A). These fractions have typically been associated with lipid rafts, which can be identified by probing for caveolin proteins^34^. Depletion of cholesterol by 5 mM MβCD had no effect on the distribution of channels, which all remained in raft-like fractions following treatment. Thus, the functional effects of cholesterol modulation we observed in human HCN channels cannot be attributed to a redistribution of channels in the cell membrane.

Next we examined the effect of altered cholesterol content on trafficking of human HCN channels to the plasma membrane. Intriguingly, biotinylation experiments indicated that the effect of cholesterol on HCN channel trafficking is specific to the isoform examined. Expression of HCN1 channels at the plasma membrane increased when cholesterol was enriched or depleted, while HCN2 trafficking was reduced with cholesterol enrichment, and unchanged by cholesterol depletion. Fewer HCN4 channels were observed at the plasma membrane upon cholesterol depletion, but increased expression compared to control was observed with cholesterol enrichment. Thus, cholesterol has isoform specific effects on HCN channel trafficking.

### Cholesterol regulation of non-equilibrium behaviour in HCN channels

**Figure 5:**
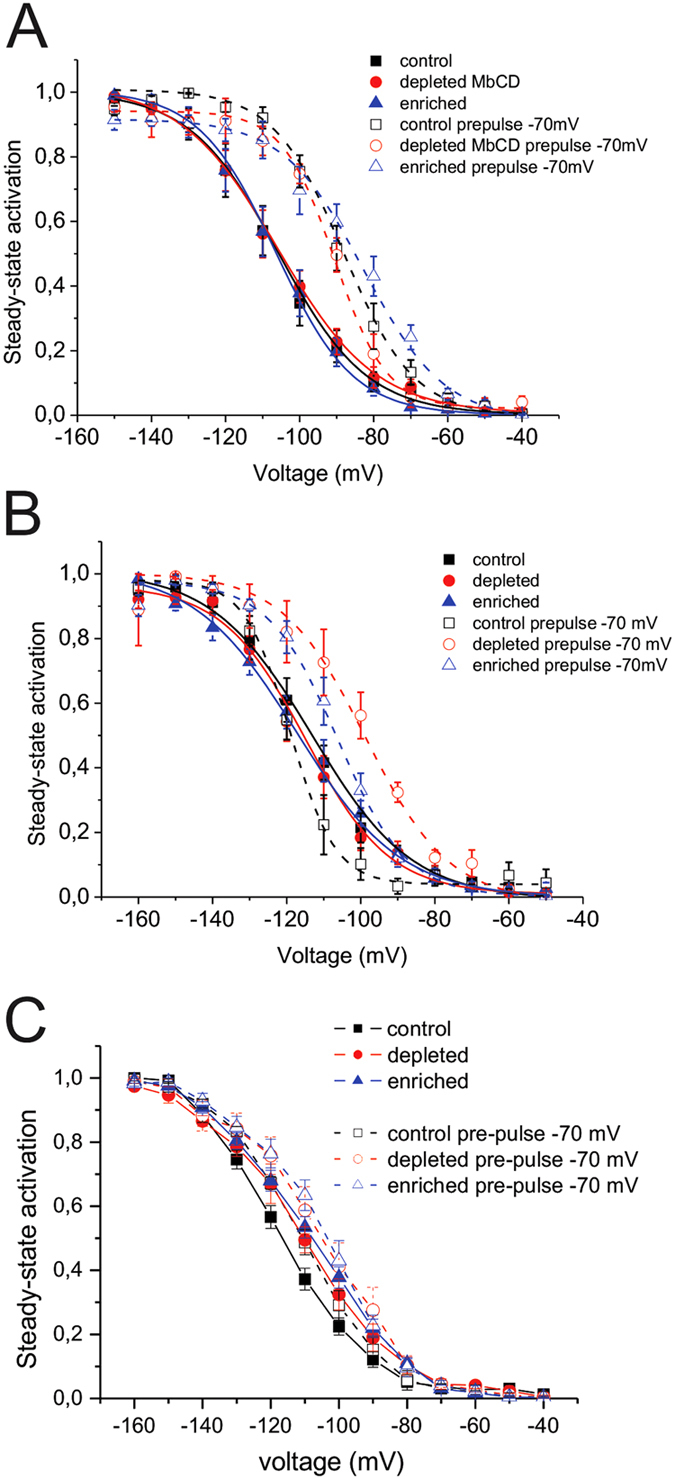
Cholesterol effects on HCN channel mode-shift (hysteresis) behaviour. (**A**) Steady-state activation of HCN1 channels with and without a 100 ms pre-pulse to −70 mV. The pre-pulse induced a +14 mV shift in the V_1/2_ of activation in HCN1 channels which was unchanged by the depletion or enrichment of membrane cholesterol. (**B**) Steady-state activation of HCN2 channels with and without a 250 ms pre-pulse to −70 mV. The pre-pulse induced did not shift the V_1/2_ of activation in HCN2 channels under control conditions, however, depletion of cholesterol induced a +10–17 mV shift in V_1/2_ of activation , while enrichment induced a +11 mV shift in V_1/2_. (**C**) Steady-state activation of HCN4 channels with and without a 1s pre-pulse to −70 mV. The pre-pulse induced a +10mV shift in the V_1/2_ of activation in HCN4 channels under control conditions, while cholesterol enrichment induced an additional +5 mV shift in V_1/2_. Cholesterol depletion did not have a large effect on HCN4 activation.

HCN channels have been shown to undergo a hysteresis in their voltage-dependence or mode-shift in which the voltage sensitivity of gating charge movement depends on the previous state^35^,^36^,^37^. Moreover, since voltage-hysteresis is thought to play an important role in preventing arrhythmias^35^,^36^,^37^, we examined the effect of cholesterol modulation on this non-equilibrium property of the cardiac HCN isoforms. Two protocols were chosen to assess hysteresis. The addition of a short −70 mV pre-pulse prior to the activation pulses, and bidirectional ramps from 0 mV to −150 mV and back. As previously reported, HCN1 channels show a large depolarizing shift in their steady-state activation in response to a −70 mV pre-pulse (Fig. 5A). Pre-pulses induced an identical shift in the V_1/2_ of activation in cells that underwent cholesterol depletion by MβCD or mevastatin, as well as cholesterol enrichment by MβCD/cholesterol (Fig. 5A). Subtle effects on the slope were observed, suggesting co-operativity may be slightly affected. On the other hand HCN2 channels did not show a shift in steady-state activation following a −70 mV pre-pulse (Fig. 5B). However, depletion of membrane cholesterol strongly shifted V_1/2_ by  +10 mV with mevastatin treatment and +17 mV with MβCD, while the slope constants remained unchanged. Enrichment of membrane cholesterol also shifted the voltage-dependence of activation by +10 mV. The −70 mV pre-pulse induced a +10 mV shift in steady-state activation in HCN4 channels. HCN4 channels that underwent treatments of cholesterol enrichment and depletion had V_1/2_’s more depolarized than control, however, −70 mV pre-pulses further shifted the V_1/2_ of HCN4 channels in membranes enriched with cholesterol (Fig. 5C).

**Figure 6:**
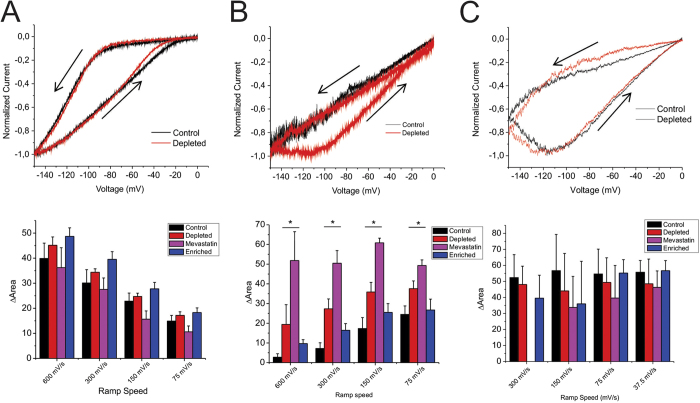
Cholesterol effects on HCN channel mode-shift (hysteresis) behaviour examined by ramps. Ramps were run from V_H_ = 0 to −150 mV and back to 0 mV at varying rates. The degree of hysteresis was quantified by the area between the forward and backward current traces. (**A**) HCN1 channels showed increasing hysteresis with faster ramp speeds, but cholesterol modulation had no effect on this behaviour. (**B**) Hysteresis could only be observed in HCN2 channels as ramp speeds slowed. However, upon membrane depletion, hysteresis could be readily observed at fast ramp speeds (eg. 600 mV/s). Cholesterol enrichment had no significant effect on hysteresis compared to HCN2 control. (**C**) Hysteresis was uniformly observed in HCN4 channels at all ramp speeds, with no observable effects from cholesterol enrichment or depletion with MβCD. Currents were too small to assess ramps at 300 mV/s for cells depleted of cholesterol by 30 μM Mevastatin. (n > 4 for all conditions; *P < 0.05).

We further examined voltage hysteresis in HCN channels using ramp protocols of various rates between 600 mV/s and 37.5 mV/s. We quantified the degree of hysteresis by the difference in area under the curves in the forward and reverse direction. We observed that for HCN1 channels, hysteresis increased with more rapid ramp speeds, and that manipulation of cholesterol levels in the membrane had no effect on the amount of hysteresis (Fig. 6A). On the other hand, HCN2 channels showed increased hysteresis as ramp speeds slowed. Cholesterol enrichment had no statistically significant consequences on the amount of hysteresis we observed for HCN2 channels, however, cholesterol depletion by MβCD and particularly mevastatin resulted in a drastic increase in the amount of hysteresis HCN2 channels underwent (Fig. 6B). This was particularly evident at 600 mV/s ramps in which in control membranes the current from 0 mV to −150 mV overlapped with the trace from −150 mV to 0 mV, while a large separation could be observed in all traces of cholesterol depleted cells expressing HCN2. Lastly, hysteresis was observed for HCN4 channels, however, the degree was unaffected by changes in ramp speeds. Moreover, hysteresis in HCN4 channels were unaffected by modulation of cholesterol levels (Fig. 6C). Taken together, these data indicate that cholesterol modifies hysteresis properties in HCN channels in an isoform specific manner.

## Discussion

In the conduction system of the heart, HCN4 channels are the predominant isoform expressed, accounting for nearly 80% of I_h_. The isoform which contributes most to the remaining current is species dependent, with HCN1 dominant in rabbit^4^ and HCN2 dominant in mouse^7^. In non-conduction tissue, HCN2 appears to be the dominant isoform with ubiquitous distribution in atrial and ventricular myocytes at low levels compared to the conduction system. Since cholesterol regulates a variety of ion channels important for cardiac function^29^,^38^,^39^,^40^,^41^, including rabbit HCN4 in which cholesterol depletion had previously been shown to modulate voltage dependence of activation and the kinetics of deactivation^28^, we systematically examined the role of cholesterol in regulating the 3 human cardiac isoforms of HCN channels. Intriguingly, we observed isoform specific differences in the regulation of these channels. While cholesterol depletion or enrichment had no effect on the voltage-dependence of human HCN1 and HCN2 activation, we observed a +10 mV shift to more depolarized potentials in human HCN4 channels. In HCN1 channels, cholesterol depletion slowed the slow-component of activation (τ_slow_), but did not alter the deactivation kinetics. Cholesterol modulation did not affect the activation kinetics of HCN2 and HCN4 channels, but cholesterol enrichment slowed the rate of deactivation in these isoforms. The effect of cholesterol modulation on channel trafficking was striking (Fig. 4B). While HCN1 channel expression increased with cholesterol depletion, the slower activation kinetics and unchanged deactivation kinetics explain the unchanged current density compared to control (Fig. 1). In HCN2 channels, cholesterol enrichment caused a reduction in surface expression, however, channels at the surface deactivated more slowly, which likely recovers the current density to control levels (Fig. 2). Cholesterol depletion did not change the expression of channels at the surface, but decreased the current density despite no changes in kinetics. It is possible that cholesterol depletion in HCN2 channels causes a reduction in the unitary conductance, or generates a subpopulation of channels that are “silenced” (ie. Popen = 0), similarly to what is expected to occur in Kir2 channels upon cholesterol enrichment^29^,^42^. This could arise from altered sensitivity to tonic levels in cAMP, or sensitivities to changes in the physiochemical properties of membranes with decreased cholesterol^43^. However, it is not immediately clear why slowed deactivation and increased expression of HCN4 channels upon cholesterol enrichment does not lead to increased current densities at steady-state.

Also striking was the isoform specific effect on hysteretic behaviour in HCN channels. By including a short −70 mV pre-pulse prior to activation, the voltage-dependence of HCN1 channel activation is more depolarized by +14 mV. Cholesterol depletion or enrichment had no effect on HCN1 hysteresis (Fig. 5), which was corroborated by ramp protocols (Fig. 6). On the other hand, HCN2 channels showed no hysteresis in untreated cells, however, cholesterol depletion shifted the voltage-dependence of activation following the pre-pulse by +10–17 mV. Cholesterol enrichment also affected hysteresis in HCN2 channels with a small depolarizing shift observed (Fig. 6). Again these results were corroborated by examining hysteresis using ramp protocols in which we observed increased sensitivity to hysteresis upon cholesterol depeletion (Fig. 6). In HCN4 channels, a pre-pulse induced at +10 mV depolarizing shift in the voltage-dependence of activation, which was furthered an additional +5 mV with cholesterol enrichment (Table 1). Cholesterol regulation appears to differentially regulate the sensitivity or propensity for hysteresis or mode-shifting to occur in each isoform. In HCN1 channels, which readily undergo mode-shifting, the manipulation of cholesterol levels does not affect hysteresis. Cholesterol appears to make it easier for HCN4 channels to undergo mode-shifting, while mode-shifting appears more readily in HCN2 channels upon cholesterol depletion. Thus, it is evident that cholesterol regulates each isoform of human HCN channels uniquely.

Mode-shifting or hysteresis in HCN channels has been suggested to play a key role in stabilizing firing rate and preventing arrhythmic firing in SAN cells and thalamic neurons^35^,^36^,^37^. Conceptually, during the hyperpolarization phase of the action potential, HCN channels do not open until very negative potentials are reached. This would prevent HCN channels from interfering with the recovery from inactivation of Na_v_ and Ca_v_ channels. On the other hand, during the interval between action potentials, HCN channels would remain open, depolarizing the membrane potential towards threshold^36^. Since hysteresis is also rate dependent, it seems as though mode-shifting in HCN channels provides additional protection against bradycardia or tachycardia. As the heart rate slows, hysteresis in HCN2 and HCN4 channels would increase, leading to more HCN current, faster depolarization and thus faster heart rate. With this in consideration, we would anticipate that excessive depletion of membrane cholesterol should increase heart rate and could trigger tachycardia. This is in line with MβCD-treated SAN cells which showed a 58% faster spontaneous rhythm^28^. Our results may in part explain why up to 1% of patients who take statins to lower cholesterol are susceptible to tachycardia. Additionally, these changes in HCN function upon cholesterol depletion may contribute to the neuropathic pain^44^ reported in approximately 9% of patients being treated with statins^45^. Alternatively, elevated amounts of blood cholesterol have also been implicated in increasing heart rate in obese patients^46^. The enhanced activity we observed for HCN2 and HCN4 channels following cholesterol enrichment could also contribute to the increased heart rate in these patients.

Cholesterol can regulate ion channels and membrane proteins by numerous mechanisms. Direct protein-sterol interactions have been reported in several ion channels, GPCRs and transport proteins^29^,^40^,^42^,^47^,^48^,^49^,^50^,^51^,^52^,^53^,^54^. These classes of proteins can also be affected by the physiochemical properties of the membrane (such as thickness, fluidity, phase, lateral pressure, etc.). Cholesterol has been shown to increase membrane thickness and decreases permeability of the bilayer^43^,^55^. Cholesterol also facilitates phase separation of lipids such as sphingolipids that contribute to the formation of lipid rafts. Consequentially, cholesterol can affect the co-localization and recruitment of other proteins or signaling pathways that regulate a specific protein of interest^56^. It has been suggested that cholesterol depletion can affect rabbit HCN4 channels in HEK293 and rabbit SAN cells by disorganizing caveolae, disrupting their interaction with caveolin-1 and caveolin-3 proteins, redistributing channels into non-raft domains and reducing overall channel expression^28^,^57^. Interestingly, ß1-adrenergic receptors also present in the SA node do not redistribute from cholesterol rich low-density fractions into higher density fractions following cyclodextran treatment^58^. In our hands, cholesterol depletion reduced the expression of HCN4 at the channel membrane (Fig. 4B) and disrupts the expression of endogenous caveolin-1 in CHO-K1 cells, however, this did not lead to a redistribution of channels into non-raft domains (Fig. 4A). While it is tempting to attribute these differences to the cell type, as HEK and SAN cells contain both caveolin-1 and caveolin-3, while CHO-K1 cells only contain caveolin-1 proteins, a more likely explanation lies in the treatments applied. In their studies, cells were treated with 1–2% MβCD for 1–2 hours, which can remove upwards of 80–90% of membrane cholesterol^59^,^60^, disrupt caveolae^59^,^60^ and may also extract significant amounts of sphingomyelin and glycosphingolipids^61^,^62^,^63^,^64^. Here we applied a milder 5 mM MβCD treatment to cells for 60 mins, which reduced the cholesterol content in lipid raft fractions by approximately 50% (Supplemental Figure 2), maintained the integrity of caveolae based on the distribution of caveolin-1 (Figure 4), and likely minimizes the removal of lipids other than cholesterol. However, despite the same effect on caveolin-1, the expression of HCN1 channels increased with cholesterol depletion and enrichment, while the expression of HCN2 channels remained the same with cholesterol depletion, and declined with enrichment. Moreover, the effects of cholesterol depletion or enrichment on gating kinetics were also isoform specific. Thus, the underlying mechanism for cholesterol regulation of HCN channels does not appear to be directly conserved among isoforms. While experiments in rabbit SAN cells suggest that cholesterol depletion by MβCD did not affect HCN4 channels by increasing basal cyclic nucleotide levels, it is yet to be determined if cholesterol alters the sensitivity to cAMP in some isoforms but not others. Our data were collected in the absence of cAMP in the pipette solution, which should enable significant rundown of cAMP levels. Thus our data is indicative of what happens in these HCN isoforms at low levels of cAMP. It also remains to be seen whether cholesterol regulation of HCN channels results from direct protein-sterol interactions, as it does in Kir channels, β-Adrenergic receptors, from changes to the properties of the bilayer, or through some intermediary protein(s).

## Conclusion

We are slowly gaining an appreciation for the regulation of ion channels and membrane proteins by the lipids in which they are embedded. We have demonstrated that cholesterol regulates the expression and function of the 3 main cardiac isoforms of HCN channels, HCN1, HCN2, and HCN4. Intriguingly, this regulation appears to be isoform specific. Further studies will be needed to address the molecular details and physiological consequences of cholesterol regulation in this family of channels.

## Additional Information

**How to cite this article**: Fürst, O. and D’Avanzo, N. Isoform dependent regulation of human HCN channels by cholesterol. *Sci. Rep.* **5**, 14270; doi: 10.1038/srep14270 (2015).

## Supplementary Material

Supplementary Information

## Figures and Tables

**Table 1:** Summary of steady-state voltage-dependence of HCN channels with and without cholesterol modulation.

**Isoform**	**Condition**	**V_1/2_ (mV)**	V_1/2_ pre-pulse(mV)	**k**	**k pre-pulse**
HCN1	Control	−102 ± 3	−88 ± 3*	10.1 ± 0.7	8.4 ± 0.9
	Depleted	−100 ± 3	−89 ± 2*,‡	11.2 ± 1.4	6.9 ± 0.6
	Mevastatin	−100 ± 6	−90 ± 8*,‡	18.1 ± 4.9	9.3 ± 0.7
	Enriched	−105 ± 3	−84 ± 4*,‡	10.5 ± 0.5	13.4 ± 2.0
HCN2	Control	−114 ± 3	−118 ± 2	11.4 ± 2.3	6.5 ± 1.5
	Depleted	−115 ± 5	−98 ± 3*,†,‡	10.4 ± 1.8	11.6 ± 2.6
	Mevastatin	−118 ± 2	−108 ± 4*,†,‡	17.8 ± 5.8	12.4 ± 1.6
	Enriched	−117 ± 4	−106 ± 2*,†,‡	14.5 ± 1.7	7.5 ± 1.0
HCN4	Control	−121 ± 4	−111 ± 2*	14.5 ± 2.0	13.2 ± 1.5
	Depleted	−111 ± 3‡	−107 ± 4‡	13.5 ± 0.7	12.3 ± 0.9
	Mevastatin	N.D.	N.D.	N.D.	N.D.
	Enriched	−108 ± 2‡	−103 ± 2*,†,‡	15.5 ± 2.2	14.2 ± 3.6

^‡^P < 0.05 vs. control no pre-pulse.

*P < 0.05 vs no-prepulse.

^†^P < 0.05 vs. control pre-pulse.
