# Supplementary material for: Isoform dependent regulation of human HCN channels by cholesterol
Source: Sci Rep. 2015 Sep 25;5:14270. doi: 10.1038/srep14270 (PMC4585891; doi:10.1038/srep14270)

Isoform dependent regulation of human HCN channels by cholesterol

Oliver Fürst and Nazzareno D'Avanzo

From the Département de physiologie moléculaire et intégrative, Université de Montréal and the Groupe d'Étude des Protéines Membranaires (GÉPROM), 2960 Chemin de la Tour, Montreal, Quebec, H3T 1J4

Address for correspondence: Ph: (514) 343-5634, FAX: (514) 343-7146,
e-mail: nazzareno.d.avanzo@umontreal.ca

Supplemental Figure 1. Cholesterol content in raft and non-raft fractions in control and treated CHO-K1 cells. Cholesterol content was quantified from CHO-K1 cell membrane fractions of 1 mL collected bydiscontinuous sucrose gradient centrifugation using an Amplex Red assay. Samples were excited at 560 nm with 20 nm bandwidth and light emission was measured at 635 nm with 35 nm bandwidth. Cholesterol content from cells treated with MβCD (red), MβCD/cholesterol (blue) and 30 μM mevastatin (magenta), were normalized to untreated control cells (black). Notably, both depletion conditions lowered cholesterol content in low-density fractions, while MβCD/cholesterol significantly enriched membrane cholesterol content in these fractions.

Supplemental Figure 2. Effects of Mevastatin treatment on HCN channels. (A) Representative traces of HCN1, HCN2, and HCN4 channels after CHO-K1 cells were treated with 30 μM mevastatin. **(B)** I-V relationships from these types of recordings indicate that mevastatin treatment resulted in a reduction of current density in each of these isoforms. **(C)** Steady-state activation curves for HCN1 and HCN2 with and without a -70 mV pre-pulse following mevastatin treatment . Instantaneous currents at +30mV were too small in HCN4 channels to reliably determine the steady-state activation values and therefore were not determined (n.d.)

Supplemental Figure 1

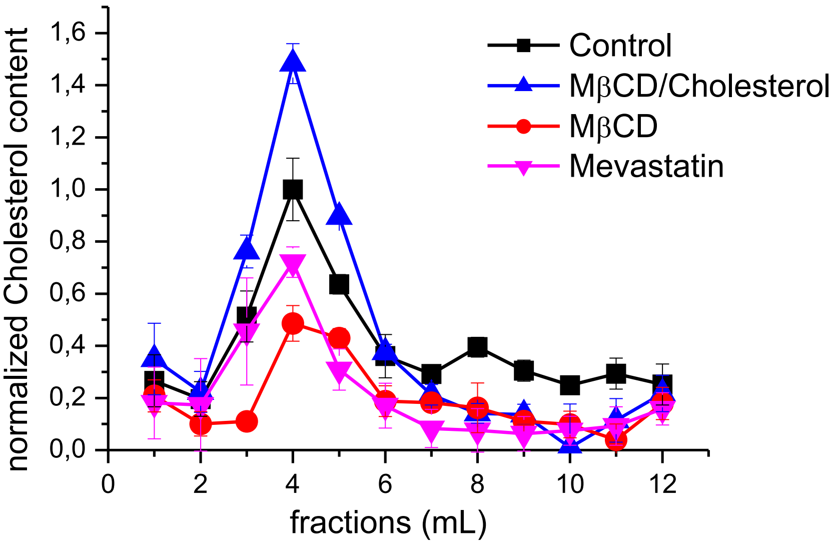

Supplemental Figure 2

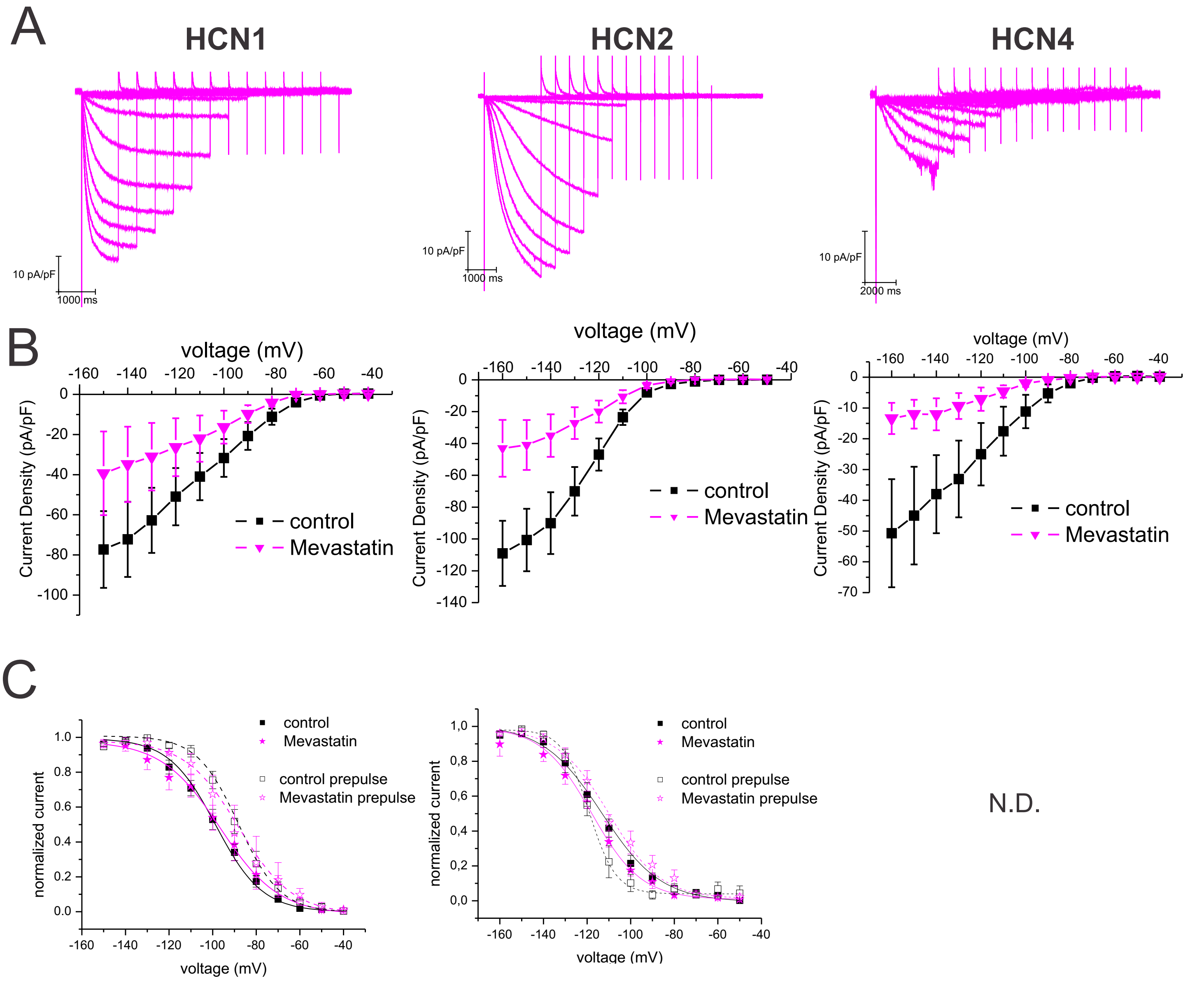

Supplement: Supplementary Information [file srep14270-s1.pdf]
